# Supplementary material for: Deficient Cardiolipin Remodelling Alters Muscle Fibre Composition and Neuromuscular Connectivity in Barth Syndrome
Source: J Cachexia Sarcopenia Muscle. 2026 Mar 17;17(2):e70246. doi: 10.1002/jcsm.70246 (PMC13140484; doi:10.1002/jcsm.70246)
Supplement: Supplementary file 1 — Figure S1: Representative single channel images for muscle fibre typing. Figure S2: Central nuclei are decreased in gastrocnemius muscles of Taz PM mice. Figure S3: Adenine nucleotide concentrations are unchanged in soleus muscles of Taz PM. Figure S4: The number of Pax7 pos cells per fibre is decreased in Taz PM muscle. Table S1: Primary antibodies. Table S2: Secondary antibodies. [file JCSM-17-e70246-s001.docx]

**Deficient Cardiolipin Remodeling Alters Muscle Fiber Composition and Neuromuscular Connectivity in Barth Syndrome**

Catalina Matias^1,2,#^, Paige L. Snider^3,#^, Elizabeth A. Sierra Potchanant^3^, Joshua R. Huot^1,2,4^, Rahul Raghav^5^, Michael T. Chin^5^, Simon J. Conway^2,3,*^, and Jeffrey J. Brault^1,2,*^

^1^Indiana Center for Musculoskeletal Health, Indiana University School of Medicine, Indianapolis, IN 46202, USA.

^2^Department of Anatomy, Cell Biology & Physiology, Indiana University School of Medicine, Indianapolis, IN 46202, USA.

^3^Herman B. Wells Center for Pediatric Research, Indiana University School of Medicine, Indianapolis, IN 46202, USA.

^4^Melvin and Bren Simon Comprehensive Cancer Center, Indiana University School of Medicine, Indianapolis, IN 46202, USA.

^5^Molecular Cardiology Research Institute, Tufts Medical Center, Boston, MA 02111, USA.

# These authors contributed equally to this work.

*Correspondence: [siconway@iu.edu](mailto:siconway@iu.edu); [jebrault@iu.edu](mailto:jebrault@iu.edu)

**Supplementary Information**

Methods

- Muscle processing for immunofluorescence staining
- Fiber Typing and centrally nucleated fibers
- Neuromuscular Junction Imaging
- Ultra-performance liquid chromatography

Tables

- Table S1: Primary antibodies
- Table S2: Secondary antibodies

Figures

- Figure S1. Representative single channel images for muscle fiber typing.
- Figure S2. Central nuclei are decreased in gastrocnemius muscles of *Taz^PM^* mice.
- Figure S3. Adenine nucleotide concentrations are unchanged in soleus muscles of *Taz^PM^*.
- Figure S4. The number of Pax7 pos cells per fiber is decreased in *Taz^PM^* muscle.

References

**Methods**

*Muscle processing for immunofluorescence staining*

Tibialis anterior (TA) and gastrocnemius muscles were excised, frozen in isopentane cooled in liquid nitrogen, and then stored at -80°C until further processing. Muscles were sectioned at -21°C using a Leica CM1950 cryostat to obtain 10 μm transverse sections of the TA for fiber typing analysis and SDH histochemistry, or 40 μm longitudinal sections of the gastrocnemius for neuromuscular junction (NMJ) imaging. Sections were mounted on microscope slides (48311-703, VWR). Slides were stored at -20°C.

*Fiber typing and centrally nucleated fibers*

Slides with transverse sections were permeabilized with 1X phosphate-buffered saline (PBS) + 0.1% Triton X-100 and blocked with a solution of 1X PBS + 0.5% BSA (BAC62, Equitech-Bio) + 10% goat serum (PCN5000, Thermo Fisher Scientific). Primary antibodies for multi-plex labeling were for Type I myosin heavy chain (MHC), Type IIA MHC, Type IIB MHC and laminin (Table S1) were diluted together in a 0.5% BSA + 2% goat serum + 1X PBS solution and incubated overnight in a humidified chamber at 4°C. The best available MHC-2B and MHC-2X primary antibodies are of the same host and isotype, preventing their use within the same multiplex. As a result, MHC-2X fibers were identified as those negative for type 1, 2A, or 2B staining. After washing with 1X PBS, the slides were incubated for one hour with the following secondary antibodies (Table S2). Post-incubation, slides were washed 3 times with 1X PBS for 5 minutes each.

For centrally nucleated fiber analysis, a slide probed only with laminin was incubated at room temperature in the dark in a 4',6-Diamidino-2-phenylindole dihydrochloride, 2-(4-Amidinophenyl)-6-indolecarbamidine dihydrochloride (DAPI) (D9542, MilliporeSigma) solution for 15 minutes, repeating the PBS washes after incubation. Slides were mounted using SlowFade Diamond Antifade Mountant (S36963, Thermo Fisher Scientific) and sealed.

Images were acquired with a Keyence BZ-X800 fluorescence microscope (Keyence Corp) at the Indiana Center for Biological Microscopy using a 10x objective. Initial image stitching and deconvolution were done in the BZ-X800 Analyzer software. The resulting images were analyzed using QuantiMus, a Flika plugin that allows for measuring the cross-sectional area (CSA), quantifying centrally nucleated fibers (CNF), and assessing the fluorescence intensity of individual myofibers.

*Neuromuscular junction imaging*

Slides with longitudinal 40-µm triceps surae tissue sections were fixed in 1% PFA (15710, Electron Microscopy Sciences) for 30 minutes and then washed with 1X PBS three times. Slides were blocked with a solution of 1X PBS + 0.3% Triton X-100 (X100, Millipore Sigma) + 1% BSA (BAC62, Equitech-Bio) + 10% donkey serum (50-588-37, Millipore Sigma) + M.O.M (MKB-2213-1, Vector Laboratories) for one hour to prevent nonspecific antibody binding.

Primary antibodies (Table S1) were diluted in a 1% BSA + 0.3% Triton-X + 1X PBS solution and then incubated overnight in a humidified chamber at 4°C. After incubation, the slides were washed in 1X PBS and incubated for two hours with α-Bungarotoxin, CF@488A (00005, Biotium) at 1:200, along with the following secondary antibodies (Table S2). Post-incubation, slides were washed with 1X PBS, mounted using SlowFade Diamond Antifade Mountant (S36963, Thermo Fisher Scientific), and sealed. All images were acquired within three days of probing.

NMJ images were acquired using a Leica SP8 Lightning confocal microscope (Leica Microsystems) equipped with a 63x glycerol immersion objective. Z-stacks were collected at 2 µm intervals, covering a total depth of 20–30 µm. Initial image stitching, deconvolution, and maximum projections were done in LAS X software. Any subsequent image processing was carried out using ImageJ.

*Ultra Performance Liquid Chromatography*

To maintain nutritive blood flow to hindlimb muscles, mice were deeply anesthetized with 2% isoflurane in oxygen delivered via a nosecone and placed on a 37 °C heating pad. Gastrocnemius and soleus muscles were excised and immediately freeze-clamped using liquid nitrogen-cooled steel clamps. Rapid freezing is essential for metabolic quenching and preservation of labile high energy phosphates. Frozen muscles were weighed to the nearest 0.1 mg. Muscles were homogenized with glass tubes and glass pestles (Kontes) at a ratio of 1 mg muscle per 49 μL extraction solution (80 % UPLC-grade methanol: 20% ultrapure water). Homogenized samples were incubated at -20°C for 30 minutes, then centrifuged at 15,000 x g for 10 min at 10°C. The pellets were discarded, and the supernatant was transferred to pre-chilled tubes.

Analytes were separated and measured using a Waters Acquity Premier UPLC system, Acquity Premier Tunable UV Detector, QDa Mass Detector, and Acquity Premier HSS T3 column with 1.8 μm VanGuard fit 2.1 × 150 mm (p/n 186009472, Waters). Chromatography buffers and conditions were developed previously to elicit baseline resolution [33, 41], which allows quantification by UV absorbance and avoids matrix effects errors. Phosphocreatine was quantified by absorbance at 210nm. Uric acid was quantified at 290nm. NADH was quantified at 338nm. NAD+, ATP, ADP, AMP, and IMP were quantified at 254nm. Identity was confirmed by mass detection with Waters QDa.

**Table S1. Primary antibodies**

| **Antibody Name** | **Host species** | **Source** | **Catalog number** | **Dilution** | **Application** |
| --- | --- | --- | --- | --- | --- |
| Laminin | Rabbit | Sigma-Aldrich | L9393 | 1:300 | IF |
| Myosin Heavy Chain 1 | Mouse | DSHB | BA-F8 | 1:100 | IF |
| Myosin Heavy Chain 2A | Mouse | DSHB | SC-71 | 1:100 | IF |
| Myosin Heavy Chain 2B | Mouse | DSHB | BF-F3 | 1:100 | IF |
| Synapsin-1 | Rabbit | Cell Signaling | 5297 | 1:200 | IF |
| β-Tubulin III | Mouse | Sigma-Aldrich | T8578 | 1:200 | IF |
| Pax7 | Mouse | DSHB | PAX7 | 1:100 | IF |
| α tubulin | Mouse | Sigma-Aldrich | T5168 | 1:1000 | WB |
| ATP5a1 | Rabbit | Proteintech | 14676-1-AP | 1:10,000 | WB |
| β-catenin | Rabbit | Abcam | ab32572 | 1:30,000 | WB |
| β tubulin | Rabbit | Abcam | ab18207 | 1:5,000 | WB |
| CHRNA1 | Rabbit | Abcam | ab308306 | 1:5,000 | WB |
| Citrate synthase | Rabbit | Cell Signaling | 14309 | 1:5,000 | WB |
| DELE1 | Mouse | Santa Cruz | Sc-515080 | 1:1,600 | WB |
| DOK7 | Rabbit | Abcam | ab75049 | 1:400 | WB |
| eIF2A, phospho-S51 | Rabbit | Abcam | ab32157 | 1:2,000 | WB |
| eIF2A, total | Rabbit | Abcam | ab169528 | 1:2,500 | WB |
| GAPDH | Mouse | Sigma-Aldrich | G8795 | 1:25,000 | WB |
| GSK3 α | Rabbit | Abcam | ab40870 | 1:30,000 | WB |
| GSK3 β | Rabbit | Abcam | ab32391 | 1:1,000 | WB |
| MCU | Rabbit | Cell Signaling | 14997 | 1:650 | WB |
| MDM2 | Rabbit | Bio-Rad | AHP1329 | 1:3,000 | WB |
| MUSK | Rabbit | Invitrogen | PA1-1741 | 1:400 | WB |
| NDUFB8 | Mouse | Abcam | ab110242 | 1:1,000 | WB |
| p53, total | Mouse | Santa Cruz | sc-71820 | 1:1,250 | WB |
| Rapsyn | Rabbit | Abcam | ab156002 | 1:5,000 | WB |
| SDHA | Mouse | Abcam | ab14715 | 1:30,000 | WB |
| SNAP25 | Rabbit | Abcam | ab109105 | 1:1,000 | WB |
| TMEM65 | Rabbit | Abcam | ab236861 | 1:2,500 | WB |
| VDAC1 | Rabbit | Invitrogen | PA1-954A | 1:80,000 | WB |

**Table S2.** **Secondary antibodies**

| **Antibody Name** | **Host species** | **Source** | **Catalog number** | **Dilution** | **Application** |
| --- | --- | --- | --- | --- | --- |
| Anti-Rabbit IgG (H+L) | Goat | Invitrogen | A-21244 | 1:500 | IF |
| Anti-Mouse IgG2b | Goat | Invitrogen | A-21140 | 1:500 | IF |
| Anti-Mouse IgG1 | Goat | Invitrogen | A-21121 | 1:500 | IF |
| Anti-Mouse IgM | Goat | Invitrogen | A-21045 | 1:500 | IF |
| Anti-Mouse IgG2a | Goat | Invitrogen | A-21143 | 1:500 | IF |
| Anti-Rabbit IgG (H+L) | Goat | Bio-Rad | 170-6515 | 1:8,000 | WB |
| Anti-Mouse IgG (H+L) | Goat | Jackson ImmunoResearch | 115-035-146 | 1:8,000 | WB |

DSHB=Developmental Studies Hybridoma Bank, IF=immunofluorescence, WB=western blot

**Figure S1**


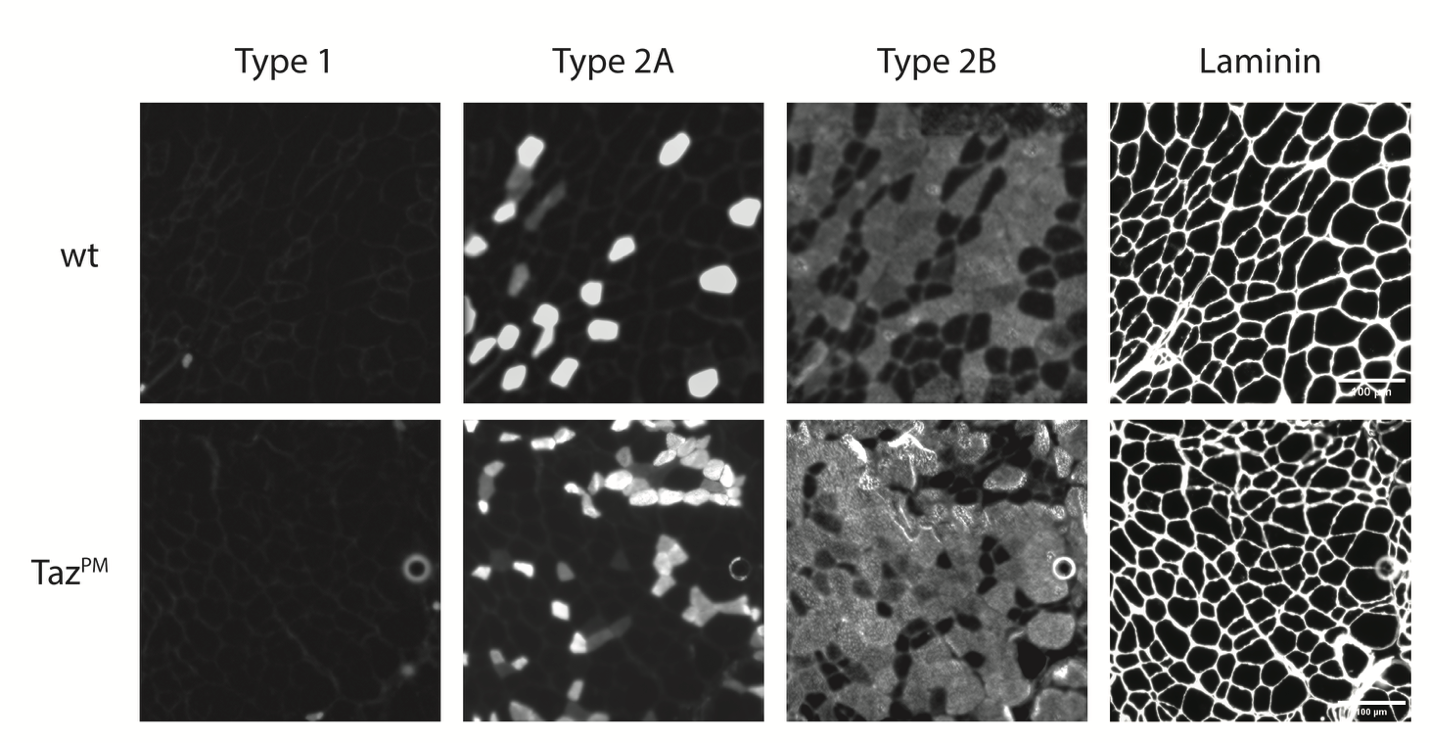


**Figure S1.** Representative single-channel fluorescent images of tibialis anterior muscle fiber typing of *wt* and *Taz^PM^* mice. Merged and pseudo-colored images are shown in Figure 1.


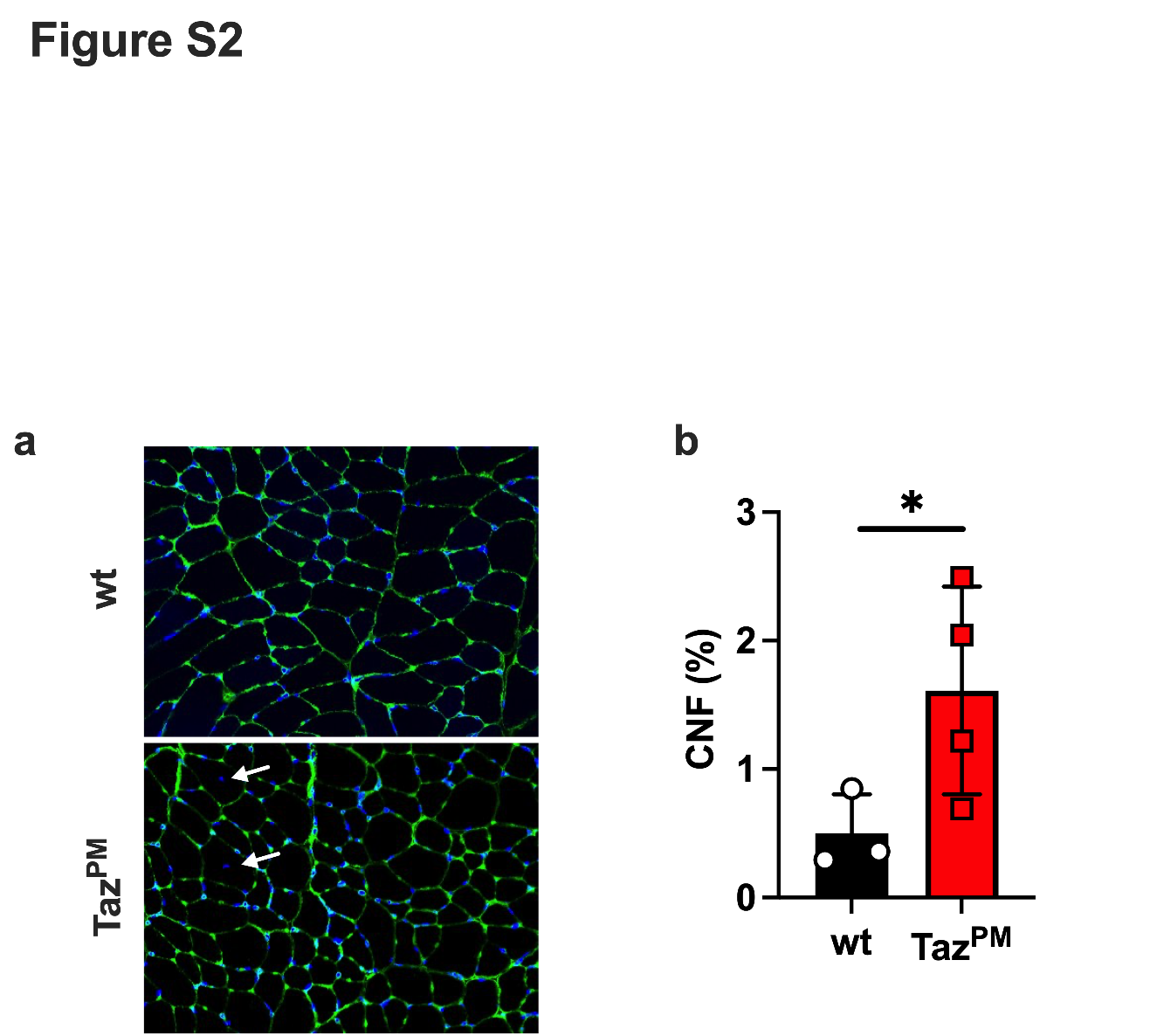


**Figure S2.** (a) Representative immunofluorescent images of gastrocnemius muscle stained for laminin (green) and nuclei (blue) in *wt* (Top) and *Taz^PM^* mice. White arrows point to central nuclei. (b) Quantification of centrally nucleated fibers is calculated as percent of fibers. n=3-4 per genotype. *p<0.05


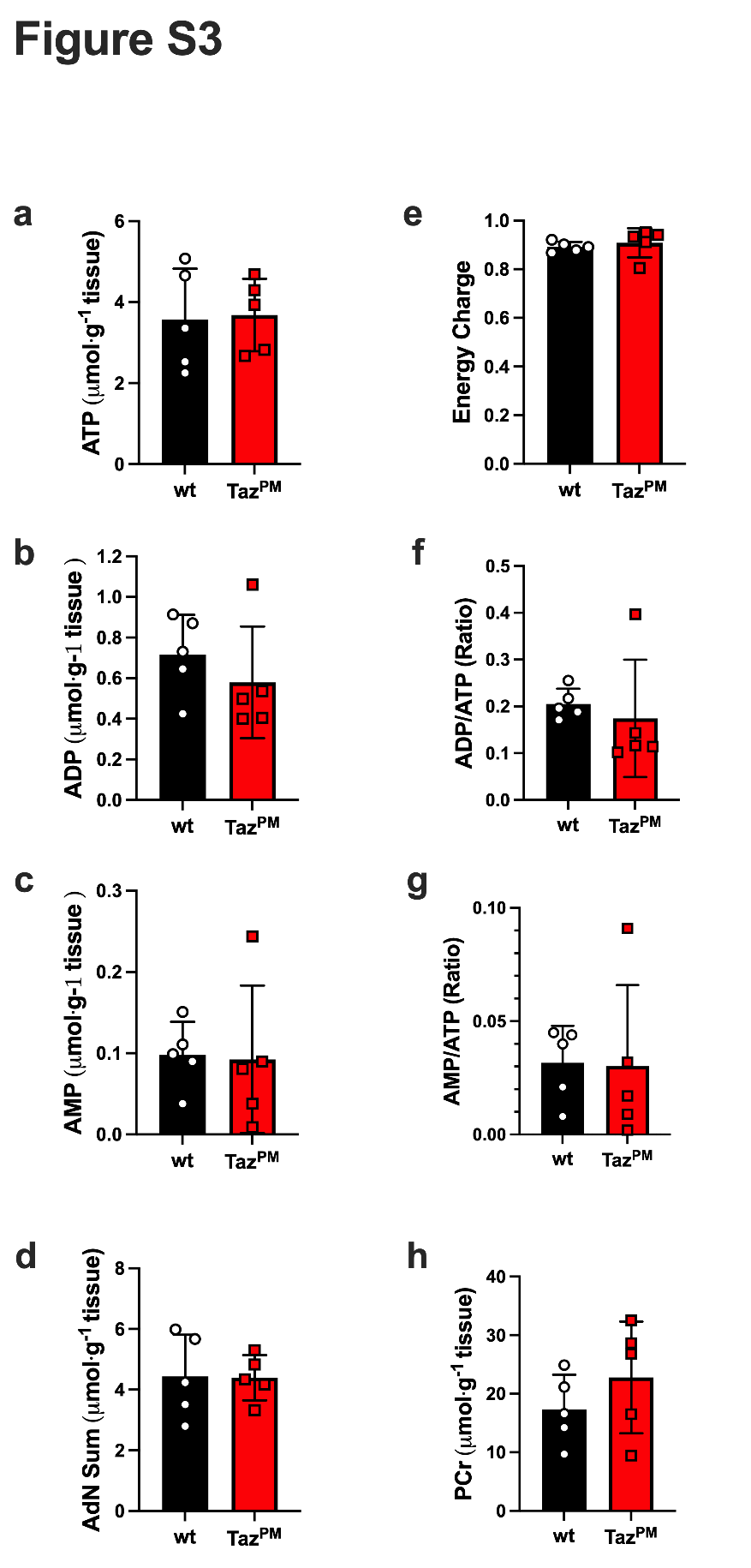


**Figure S3. Adenine nucleotide concentrations are unchanged in soleus muscles of *Taz^PM^*.** Ultra-performance liquid chromatography analysis of (a) ATP, (b) ADP, and (c) AMP in soleus muscles of *wt* and *Taz^PM^* mice. Calculations of (d) AdN Sum = ATP + ADP + AMP, (e) Energy Charge = (ATP + (0.5 * ADP)) / (ATP + ADP + AMP), (f) ADP/ATP ratio, (g) AMP/ATP ratio, and (h) phosphocreatine (PCr). n=5 per genotype.


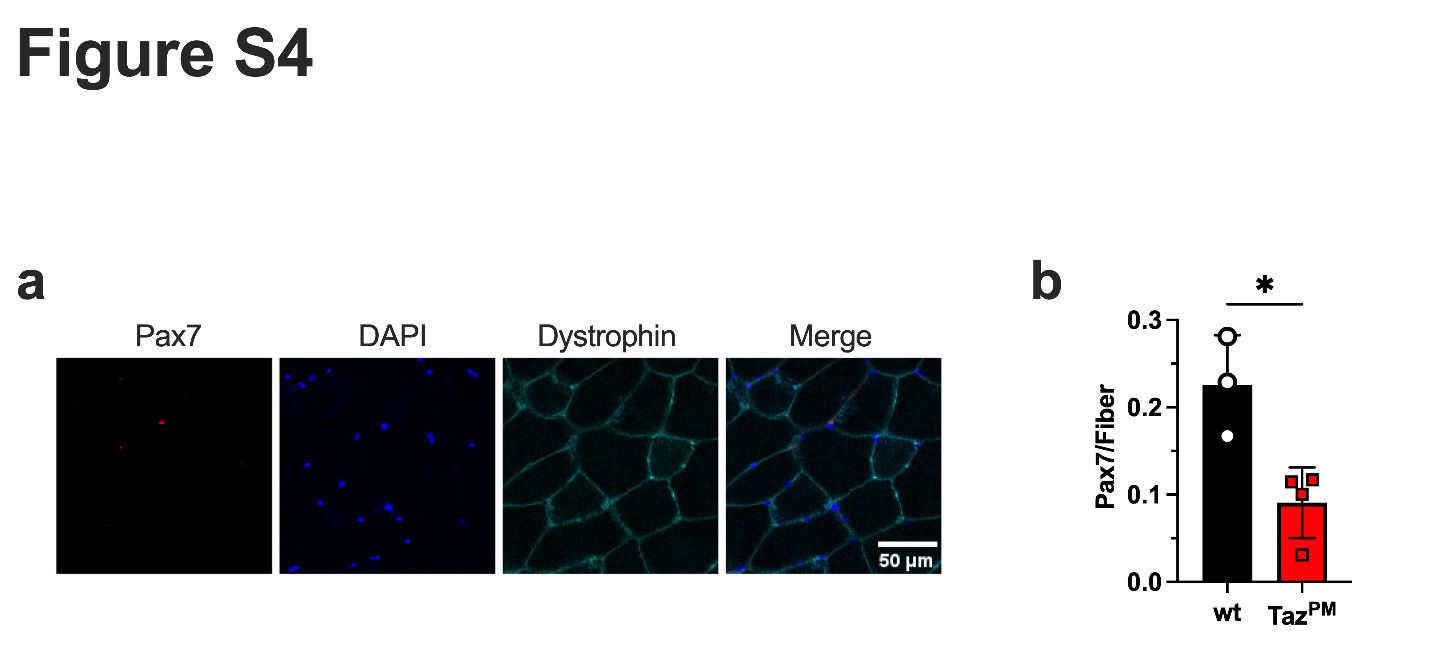


**Figure S4. The number of Pax7 positive cells per fiber is decreased in *Taz^PM^* muscle.** (a) Representative immunofluorescent images of gastrocnemius muscle stained for dystrophin (green), nuclei (blue), and Pax7 in *wt* mice. (b) Quantification of Pax7 positive cells per number of fibers. n=3-4 per genotype. *p<0.05

Supplemental References

S1. Gonzalez IL. Human TAFAZZIN variants database. Retrieved from Barth Syndrome Foundation [<https://www.barthsyndrome.org/research/tafazzindatabase.html>]. 2000.

S2. Goncalves RLS, Schlame M, Bartelt A, Brand MD, Hotamisligil GS. Cardiolipin deficiency in Barth syndrome is not associated with increased superoxide/H2 O2 production in heart and skeletal muscle mitochondria. FEBS Lett 2021;595:415-32.

S3. Snider PL, Sierra Potchanant EA, Matias C, Edwards DM, Brault JJ, Conway SJ. The Loss of Tafazzin Transacetylase Activity Is Sufficient to Drive Testicular Infertility. J Dev Biol 2024;12:

S4. Deacon RM. Measuring the strength of mice. J Vis Exp 2013;76:e2610.

S5. Chida J, Yamane K, Takei T, Kido H. An efficient extraction method for quantitation of adenosine triphosphate in mammalian tissues and cells. Anal Chim Acta 2012;727:8-12.

S6. Kastenschmidt JM, Ellefsen KL, Mannaa AH, Giebel JJ, Yahia R, Ayer RE, et al. QuantiMus: A Machine Learning-Based Approach for High Precision Analysis of Skeletal Muscle Morphology. Front Physiol 2019;10:1416.

S7. Huot JR, Pin F, Bonetto A. Muscle weakness caused by cancer and chemotherapy is associated with loss of motor unit connectivity. American Journal of Cancer Research 2021;11:2990-3001.

S8. Law AS, Hafen PS, Brault JJ. Liquid chromatography method for simultaneous quantification of ATP and its degradation products compatible with both UV–Vis and mass spectrometry. Journal of Chromatography B 2022;1206:123351.

S9. Gordon T. Reinnervated muscle fiber type-grouping-inevitable? Oncotarget 2017;8:17410-1.

S10. Martineau E, Di Polo A, Vande Velde C, Robitaille R. Dynamic neuromuscular remodeling precedes motor-unit loss in a mouse model of ALS. Elife 2018;7:

S11. Batista AFR, Martinez JC, Hengst U. Intra-axonal Synthesis of SNAP25 Is Required for the Formation of Presynaptic Terminals. Cell Rep 2017;20:3085-98.

S12. Baran R, Castelblanco L, Tang G, Shapiro I, Goncharov A, Jin Y. Motor neuron synapse and axon defects in a C. elegans alpha-tubulin mutant. PLoS One 2010;5:e9655.

S13. Valenzuela DM, Stitt TN, Distefano PS, Rojas E, Mattsson K, Compton DL, et al. Receptor Tyrosine Kinase Specific for the Skeletal-Muscle Lineage - Expression in Embryonic Muscle, at the Neuromuscular-Junction, and after Injury. Neuron 1995;15:573-84.

S14. Inoue A, Setoguchi K, Matsubara Y, Okada K, Sato N, Iwakura Y, et al. Dok-7 activates the muscle receptor kinase MuSK and shapes synapse formation. Sci Signal 2009;2:ra7.

S15. Moransard M, Borges LS, Willmann R, Marangi PA, Brenner HR, Ferns MJ, et al. Agrin regulates rapsyn interaction with surface acetylcholine receptors, and this underlies cytoskeletal anchoring and clustering. J Biol Chem 2003;278:7350-9.

S16. Oakhill JS, Steel R, Chen ZP, Scott JW, Ling N, Tam S, et al. AMPK is a direct adenylate charge-regulated protein kinase. Science 2011;332:1433-5.

S17. Jurecka A, Tylki-Szymanska A. Inborn errors of purine and pyrimidine metabolism: A guide to diagnosis. Mol Genet Metab 2022;136:164-76.

S18. Miller SG, Matias C, Hafen PS, Law AS, Witczak CA, Brault JJ. Uric acid formation is driven by crosstalk between skeletal muscle and other cell types. JCI Insight 2024;9:

S19. Covarrubias AJ, Perrone R, Grozio A, Verdin E. NAD(+) metabolism and its roles in cellular processes during ageing. Nat Rev Mol Cell Biol 2021;22:119-41.

S20. Kushmerick MJ, Moerland TS, Wiseman RW. Mammalian Skeletal-Muscle Fibers Distinguished by Contents of Phosphocreatine, ATP, and Pi. P Natl Acad Sci USA 1992;89:7521-5.

S21. Bloemberg D, Quadrilatero J. Rapid determination of myosin heavy chain expression in rat, mouse, and human skeletal muscle using multicolor immunofluorescence analysis. PLoS One 2012;7:e35273.

S22. Liu J, Xiao Q, Xiao J, Niu C, Li Y, Zhang X, et al. Wnt/beta-catenin signalling: function, biological mechanisms, and therapeutic opportunities. Signal Transduct Target Ther 2022;7:3.

S23. Wang QM, Park IK, Fiol CJ, Roach PJ, DePaoli-Roach AA. Isoform differences in substrate recognition by glycogen synthase kinases 3 alpha and 3 beta in the phosphorylation of phosphatase inhibitor 2. Biochemistry-Us 1994;33:143-7.

S24. Schiaffino S, Reggiani C. Fiber types in mammalian skeletal muscles. Physiol Rev 2011;91:1447-531.

S25. Stienen GJ, Kiers JL, Bottinelli R, Reggiani C. Myofibrillar ATPase activity in skinned human skeletal muscle fibres: fibre type and temperature dependence. J Physiol 1996;493 (Pt 2):299-307.

S26. Barclay CJ, Constable JK, Gibbs CL. Energetics of fast- and slow-twitch muscles of the mouse. J Physiol 1993;472:61-80.

S27. Karpati G, Engel WK. "Type grouping" in skeletal muscles after experimental reinnervation. Neurology 1968;18:447-55.

S28. Genin EC, Madji Hounoum B, Bannwarth S, Fragaki K, Lacas-Gervais S, Mauri-Crouzet A, et al. Mitochondrial defect in muscle precedes neuromuscular junction degeneration and motor neuron death in CHCHD10(S59L/+) mouse. Acta Neuropathol 2019;138:123-45.

S29. Fuentes JM, Morcillo P. The Role of Cardiolipin in Mitochondrial Function and Neurodegenerative Diseases. Cells 2024;13(7):609.

S30. Rizzuto E, Pisu S, Nicoletti C, Del Prete Z, Musaro A. Measuring Neuromuscular Junction Functionality. J Vis Exp 2017;

S31. Baird TD, Wek RC. Eukaryotic initiation factor 2 phosphorylation and translational control in metabolism. Adv Nutr 2012;3:307-21.

S32. Kutschka I, Bertero E, Wasmus C, Xiao K, Yang L, Chen X, et al. Activation of the integrated stress response rewires cardiac metabolism in Barth syndrome. Basic Res Cardiol 2023;118:47.
